# Correction to “Growth‐Associated Protein 43 Levels in the Cerebrospinal Fluid Correspond to the Cerebral Blood Flow Alterations in Alzheimer's Dementia Continuum: An Original Study”

**DOI:** 10.1002/hsr2.73415

**Published:** 2026-10-18

N. Songhori, M. Shamsi Goushky, M. Mojerloo, M. Ghajar, Y. Kianifar, F. Karimzadeh, F. Khosravi (2025). Growth‐Associated Protein 43 Levels in the Cerebrospinal Fluid Correspond to the Cerebral Blood Flow Alterations in Alzheimer's Dementia Continuum: An Original Study. Health Science Reports, 8(3).e70534. https://doi.org/10.1002/hsr2.70534

The following corrections have been made: the author list has been updated to remove researchers who were incorrectly listed as authors. The Alzheimer's Disease Neuroimaging Database has been added as an author, with further explanation provided in the Acknowledgements. The authors have provided new details on the units and values in Table 1. Individual level data has been removed from Supporting Data, in line with the ADNI guidelines on data sharing. The changes are explained in detail below.

The corrected author list is: N. Songhori, M. Shamsi Goushky, M. Mojerloo, M. Ghajar, Y. Kianifar, F. Karimzadeh, F. Khosravi.

The investigators at the Alzheimer's Disease Neuroimaging Initiative (ADNI) database, who contributed to the design and implementation of ADNI and/or provided data, but did not participate in the analysis or writing of this report, have been added as authors. The following statement has been added to the Acknowledgements' section: “*Data used in preparation of this article were obtained from the Alzheimer's Disease Neuroimaging Initiative (ADNI) database (adni. loni. usc. edu). As such, the investigators within the ADNI contributed to the design and implementation of ADNI and/or provided data but did not participate in the analysis or writing of this report. A complete listing of ADNI investigators can be found at:* http://adni.loni.usc.edu/wp-content/uploads/how_to_apply/ADNI_Acknowledgement_List.pdf.”

The individual‐level data has been removed from the Supporting Information, in accordance with the guidelines on data sharing from the ADNI database. The summary‐level (i.e., not participant‐level) data has been retained.

Finally, regarding Table 1, the table itself is correct and provided below:

Table 1:

The authors have added this note to clarify that: Continuous and categorical variables are presented as mean ± standard deviation and number (percentage), respectively. The Chi‐square test was used for dichotomous variables. Bold format represents significant values.

Abbreviations: ASL, arterial spin label; MMSE, mini mental state exam. a Indicates Mann‐Whitney test; otherwise, Independent T‐test p value is presented for continuous variables. The unit of measurement for Mean CBF in Table 1 is “ml/100 g tissue/min” (milliliters per 100 grams of tissue per minute).

